# Knowledge and lifestyle modifications for dyslipidaemia among patients on statins in Pretoria

**DOI:** 10.4102/jcmsa.v2i1.25

**Published:** 2024-04-09

**Authors:** Thandazile Z. September, John V. Ndimande, Henry I. Okonta, Carien Steyn, Tombo Bongongo

**Affiliations:** 1 Department of Family Medicine and Primary Health Care, School of Medicine, Sefako Makgatho Health Sciences University, Pretoria, South Africa

**Keywords:** dyslipidaemia, knowledge, practices, lifestyle modifications, primary healthcare, Pretoria or Tshwane District, South Africa

## Abstract

**Background:**

Nearly half of all non-communicable diseases are caused by cardiovascular diseases, for which one of the most important risk factors is dyslipidaemia. This study assessed the knowledge and lifestyle modification practices regarding dyslipidaemia among patients taking statins at Phedisong 4 Community Health Centre in Pretoria, South Africa.

**Methods:**

This was a descriptive cross-sectional study using a structured administered and piloted questionnaire.

**Results:**

The mean age of the 268 participants was 60.1 years, with a minimum and maximum of 27 years and 89 years, respectively. High participation rates were seen among females (70.2%), those who were overweight (44.8%), adults with high school level education (48.1%), pensioners (54.1%), those with low incomes (83.2%), non-smokers (86.9%), and people with co-morbid conditions, including hypertension (92.2%) and diabetes mellitus (31.0%). Findings revealed that 66.3% of participants with dyslipidaemia adjusted their lifestyles, despite poor knowledge. There was a significant relationship between poor knowledge and poor practices in 55.6% of the participants (*p* < 0.001).

**Conclusion:**

This study found that participants’ lack of information about dyslipidaemia did not affect their behaviour because more than half of them practiced lifestyle modifications, which can be related to the study sample’s over 90% concomitant hypertension diagnosis. Ongoing education on dyslipidemia should be addressed through a variety of venues, including health education at different health institutions.

**Contribution:**

It is necessary to maintain ongoing education of patients about dyslipidaemia, its treatment, and related lifestyle modifications through a variety of channels, including health education in different health facilities around Pretoria.

## Introduction

Cholesterol is produced in the liver and comes from animal sources (foods), and is essential in order to produce numerous hormones and bile for digestion.^1^ A high total cholesterol level in the blood is referred to as hyperlipidaemia, and a normal value is less than 5 mmol/L.^1,2^ Different types of cholesterol, including low-density lipoprotein (LDL) cholesterol, triglycerides and high-density lipoprotein cholesterol (HDL-C) form part of the total cholesterol.^1^ Dyslipidaemia is a more favourable term than hyperlipidaemia, because it also includes a decreased concentration of HDL-C.^3^

Dyslipidaemia, including hypercholesterolaemia can be categorised according to aetiology as either primary/familial or secondary.^4^ Primary hypercholesterolaemia is an autosomal-dominant genetic condition with a predominant elevation of LDL cholesterol.^4,5,6^ Lifestyle variables such as weight gain, obesity, excessive alcohol consumption and other factors, such as medical problems (hypothyroidism, diabetes mellitus, etc.), and medications like protease inhibitors are secondary causes of dyslipidaemia.^4,7^ Both primary and secondary hyperlipidaemia have the same management: lifestyle modifications and pharmacological means such as statins as the first-line drug treatment.^2,7,8^ If not managed, dyslipidaemia predisposes the individual to cardiovascular diseases, especially elevated LDL.^9^

Dyslipidaemia, obesity, type 2 diabetes and hypertension are all chronic lifestyle disorders that increase the risk of developing cardiovascular diseases, and their frequency is rising in South Africa.^10,11^ Research shows that westernisation of the African diet and lifestyle has contributed to increased risk of developing metabolic and non-communicable conditions.^12^

It was estimated by the World Health Organization (WHO) that almost 40% of the global population has dyslipidaemia, and 22% of that total lives in Africa.^13^ Community-based surveys in South Africa have reported the prevalence to be up to 90% in some cases.^2,7,14,15^ The prevalence of dyslipidaemia is even higher in patients with other chronic non-communicable conditions, especially those with diabetes mellitus.^2,11,15^

There have been several studies that show the level of disease awareness among patients in the general population and in healthcare institutions, with limited local data. In India, more than half (57.0%) of the patients on hypolipidaemic medications at the Rajiv Gandhi Institute of Medical Sciences were unaware that they had hyperlipidaemia, supporting research from Beijing, China, conducted 7 years earlier which found 56% of participants were unaware of their condition.^16,17^ This contrasts with general population studies conducted in 2014, where only 4% of patients had awareness of their condition in the South African HAALSI study.^14^

There is a paucity of previous studies conducted to assess the patient’s knowledge about dyslipidaemia, both in the global and local spheres. In an Indian study, one-third of the participants did not understand the chronicity of taking hypolipidaemics, whereas in an American study, participant with both hypertension and dyslipidaemia were not aware of the effects of dyslipidaemia.^17,18^ In South Africa, however, most participants (81%) stated that nutrition and weight loss are the main components of treatment of dyslipidaemia, and obesity and dyslipidaemia awareness were highly correlated with being overweight or obese.^14^

The South African Heart Association and the Lipid and Atherosclerosis Society of Southern Africa have developed guidelines for lifestyle modifications for patients with dyslipidaemia.^7^ However, to be sustainable, dietary advice and exercise must be practical and tailored to the patient’s personal and cultural preferences.^19^ Smoking cessation, reaching and maintaining a goal weight, and alcohol moderation are also included in the lifestyle modification guidelines.^7,20^

In a study conducted in 2005 in Beijing it was noted that that the longer the patients had dyslipidaemia for, the more lifestyle adjustments they made, such as adopting healthier diets and stopping smoking.^16^ Patients who are identified as being at high risk for dyslipidaemia development can be given non-pharmacological or lifestyle modification advice/education as part of primary prevention, which is tailored for each patient.^21^ A Kenyan study looking at factors associated with dyslipidaemia in diabetic patients revealed that while 99% of the participants received dietary advice to manage their condition, only 62% adhered to that advice, and 37% were adherent to taking their medication as prescribed, with 68% regularly attending the clinic.^22^ Therefore, in this study there was poor adherence to medication and dietary advice, despite the patients receiving education on dietary management; however, exercise adherence was satisfactory.^22,23^ A multi-centre study conducted in two South African provinces (Eastern Cape and Mpumalanga) showed that only half of the patients were physically active, and 97% consumed fruit and vegetables 1–3 times per week; however, 75% still consumed fast food 1–3 times per week and over 85% used vegetable oil.^2^

Some factors have been identified as linked to non-adherence and poor compliance with advice on lifestyle changes, which include patient and clinical factors. These factors include a complex regimen of lifestyle changes, poor cognition, lack of patient motivation and adverse effects of the proposed regimen (e.g., musculoskeletal pain after exercise).^21^

Nutritional modifications that have been shown to reduce the long-term risk of cardiovascular disease are the Dietary Approaches to Stop Hypertension (DASH) and the Mediterranean diet.^7^ Exercise, particularly high-intensity intermittent aerobic exercises, has been associated with a reduction in triglycerides by up to 40%, an increase in HDL-C of up to 20%, and an overall reduction in cardiovascular risk.^24^

The aim of this study is to assess the demographics of patients with dyslipidaemia who are taking statins, and to measure their knowledge and practices regarding their condition in a Pretoria primary healthcare setting.

## Research methods and design

### Study design

This was a descriptive cross-sectional study using a structured, administered and piloted questionnaire.

### Study setting

The study was conducted at Phedisong 4 Community Health Centre (CHC), which is in Ga-Rankuwa, Zone 4 Township of the Tshwane District in Gauteng province, South Africa. Ga-Rankuwa is a sizeable township located about 37 km to the northwest of Pretoria.

### Study population, sampling technique and sample size

In the ‘chronic diseases’ section of Phedisong 4 CHC, although the facility services around 57 000 people, the attendance patterns over a 3-month period showed that on average 800 patients were seen each month. By utilising the Cochran sample size formula downloaded from the internet, a sample size of 260 was obtained, with a 95% confidence interval, a 5% margin of error, and a population of 800.^25^ Convenience sampling yielded a final sample size of 268, when all patients who agreed to take part in the study and met the inclusion criteria during the data collection period were included. Those who were included had to be known patients of Phedisong 4 CHC, 18 years of age or above, with dyslipidemia in the medical record, and on statins. Patients who had cognitive impairment were not included.

### Data collection

The researcher trained two research assistants who are multilingual and able to speak and write in English, Setswana, and IsiZulu as the dominant languages in the area. They were trained on how to present the study and its aim and objectives to potential participants in the chronic diseases section of the CHC. They were also tasked with assisting participants who had signed consent forms with filling in of questionnaires in the language of preference, as it was available in both English and Setswana. Both the questionnaire and the consent forms were marked with ascending numbers as identifiers. Patients were verbally asked if they had participated in the study before, to avoid re-recruiting.

The information in the study questionnaire was extracted from the South African Dyslipidaemia Guideline Consensus Statement as well as from the literature review.^7^ Based on that information, the questionnaire was compiled by the researcher, with questions divided into three groups: sociodemographic characteristics, knowledge, and practice of lifestyle modification.

Prior to the pilot study, the questionnaire underwent retest reliability testing to determine the tool’s reliability. This entails administering the identical questionnaire to four patients at another CHC (Temba CHC), which is located 221 km away from Phedisong 4 CHC, where the study will be conducted. After 2 weeks, the same four patients were given the same questionnaire, and the results were comparable to the first survey. This questionnaire was also evaluated by three family physicians with an interest in research to determine whether the material was appropriate for the aim (face validity).

*Pilot study*: The questionnaire was exposed to a pilot study prior to the main study. Nine patients who met the inclusion criteria took part. The study was conducted in a clinic about 70 km from the main clinic of the study. After the pilot study, the questionnaire was adjusted by the researcher, and thereafter it was assessed and adjusted by four supervisors. It was tested for reliability using test–retest reliability, and for validity using face validity.

Data collection took 11 weeks, from October 2019 to December 2019.

### Data analysis

Raw data from the questionnaire were captured on an Excel spreadsheet before it was imported into SAS software version 9.4 (SAS Institute Inc, Cary, NC, United States), where descriptive analysis was used to analyse the data. The results, provided according to frequency and percentages, are presented in tables. The Fisher Exact test and Pearson Chi-square test were used to determine the significance of the relationship between variables, which was expressed as a *p*-value, with a *p*-value of 0.05 being significant. Unfortunately, no meaningful correlations emerged.

### Ethical considerations

Ethical clearance to conduct this study was obtained from the Sefako Makgatho Health Sciences University Research Ethics Committee (No. SMUREC/M/03/2019: PG), and the provincial or Gauteng registration number of this study was obtained through Tshwane Research Committee (No. GP_201907_034). Before exposing participants to the study questionnaire, a signed informed consent form was obtained. A strict policy of confidentiality and anonymity was followed throughout the entire study. Participants were made aware of their right to leave the study process at any time, should they wish to do so. Data were kept on a password protected computer with access only to the main author.

## Results

The majority of the participants were over the age of 49 years (78.4%), female (70.2%), pensioners (54.1%), had secondary school level education (48.1%), earned below the living wage (83.2%), had hypertension (92.2%), were overweight/obese (72.8%), and non-smokers (86.9%). Of those who smoked (*n* = 35), the majority had a significant smoking history (62.9%) (Table 1).

**TABLE 1:** Sociodemographic characteristics of participants.

Sociodemographic variables	Frequency	
	*n*	%
**Age (years)**		
20–29	3	1.1
30–39	14	5.2
40–49	41	15.3
50–59	58	21.6
60–69	87	32.5
70–79	60	22.4
80–89	5	1.9
Total	268	100.0
**Gender**		
Male	80	29.8
Female	188	70.2
Total	268	100.0
**Body mass index (BMI) (kg/m^2^)**		
Normal: 18.5 to < 25.0	73	27.2
Overweight: 25.0 to < 30.0	120	44.8
Obese: ≥ 30.0	75	28.0
Total	268	100.0
**Education level**		
None	8	3.0
Primary level (Grades 0–5)	59	22.0
Secondary level/high school (Grades 6–10)	129	48.1
Matric completed	50	18.7
Tertiary level/college	22	8.2
Total	268	100.0
**Employment status**		
Unemployed	61	22.8
Temporarily employed	18	6.7
Permanently employed	33	12.3
Self-employed	11	4.1
Pensioner	145	54.1
Total	268	100.0
**Income (R) per month**		
< 3000.00	223	83.2
3500.00 – 10 000.00	39	14.6
> 10 000	6	2.2
Total	268	100.0
**Smoker**		
Yes	35	13.1
No	233	86.9
Total	268	100.0
**Pack years (cigarettes/day/year)**		
1–10	13	37.1
> 10	22	62.9
Total	35	100.0
**Co-morbidities**		
Hypertension	247	92.2
Diabetes	83	31.0
Cardiovascular disease (heart failure, cardiomyopathy, ischaemic heart disease)	9	3.4
None	1	0.4
Total	340	-

Each participant responded to 12 items in the questionnaire as presented in Table 2. For each question, an affirmative response was marked as a good answer, corresponding to ‘Yes’; all negative responses were marked as inappropriate or poor answers, corresponding to ‘No’, and unsure responses were marked as ‘Not sure’. The total of all ‘Yes’ responses per question was 1464, corresponding to 44.1% of all responses. On the other hand, responses ‘Not sure’ and ‘No’ were grouped to indicate that the response was inadequate. There were 1400 (42.2%) ‘No’ responses and 451 (13.6%) ‘Not sure’ responses.

**TABLE 2:** Assessment of participants’ knowledge about dyslipidaemia.

Questions about their knowledge		Responses					
		Yes		No		Not sure	
		*n*	%	*n*	%	*n*	%
1.	Do you know foods that can cause high cholesterol level in the blood?	155	57.8	30	11.2	83	30.9
2.	Being overweight does not mean that you have high cholesterol.	120	44.7	130	48.5	18	6.7
3.	Does being overweight increase your chances of having dyslipidaemia?	151	56.3	100	37.3	17	6.3
4.	Is dyslipidaemia a treatable condition?	70	26.1	177	66.0	21	7.8
5.	Is there something you can do at home to decrease cholesterol levels?	104	38.8	132	49.2	32	11.9
6.	Do you know the normal level of total cholesterol?	26	9.7	172	64.1	70	26.1
7.	Do you know any source of ‘good’ fat/oil/cholesterol?	9	3.3	249	92.9	109	3.75
8.	Do you know any health benefits of lowering cholesterol?	111	41.4	140	52.2	17	6.3
9.	Do you think high cholesterol can lead to high blood pressure?	198	73.8	40	14.2	30	11.1
10.	Do you think high cholesterol can lead to a heart attack?	245	91.4	11	4.1	12	4.4
11.	Has your cholesterol lowered since starting anti-cholesterol medication?	213	79.4	30	11.1	25	9.3
12.	Do you know how long you will have to take anti-cholesterol medication for?	62	23.1	189	70.5	17	6.3

**Total responses (% based on the total responses)**		**1464**	**44.1**	**1400**	**42.2**	**451**	**13.6**

This study revealed that 55.8% of the responses the participants had showed poor knowledge about dyslipidaemia, when considering the responses of ‘No’ and ‘Not sure’ to be inappropriate answers (Table 2).

For each participant in the study the number of correct answers was expressed as a percentage out of 12 and rated as *Poor knowledge (< 50%), Average knowledge (50%* to *< 75%)*, or *Good knowledge (≥ 75%)*, for the patient. The overall knowledge distribution for all 268 participants is presented in Table 3.

**TABLE 3:** Overall knowledge distribution.

Knowledge (%)	*n*	%
Poor knowledge (< 50)	126	47.0
Average knowledge (50 to < 75)	133	49.6
Good knowledge (≥ 75)	9	3.4

**Total**	**268**	**100**

The study found that participants had 66.3% of responses indicating good global practices of lifestyle modification. However, those who smoked had no plans to stop (Table 4).

**TABLE 4:** Assessment of lifestyle modification practices among participants.

Questions on lifestyle modification		Responses							
		Always		Sometimes		Never		Not sure	
		*n*	%	*n*	%	*n*	%	*n*	%
1.	How often do you take your cholesterol-lowering medication?	233	87.00	25	9.0	9	3.30	1	0.30
2.	Have you made any changes in your diet since high cholesterol diagnosis?	202	75.00	66	25.0	0	0.00	0	0.00
3.	Do you eat vegetables daily?	151	56.00	109	41.0	2	0.74	6	2.20
5.	Do you exercise daily?	146	54.00	106	40.0	16	6.00	0	0.00
6.	If you smoke, do you have plans to stop smoking?†	0	0.00	26	74.2	8	22.90	1	2.90
7.	Do you think you have enough information to help you manage your cholesterol?	180	67.00	84	31.0	3	1.10	1	0.30

**Total responses**		**912**	**66.30**	**416**	**30.2**	**38**	**2.70**	**9**	**0.09**

† , Thirty five participants are smokers.

For each patient in the study, the number of ‘Priority lifestyle modifications’ was expressed as a percentage out of seven and rated as *Poor modifications (< 50%), Average modifications (50% – < 75%),* or *Good modifications (*≥ *75%),* for the patient. The overall distribution for all 268 patients is displayed in the Table 5. Modifications are classified as poor (< 50%), average (50% to < 75%), or good (≥ 75%) for patients. There were more participants in the category ‘average modifications’ (56%).

**TABLE 5:** Overall lifestyle modifications distribution.

Categories	*n*	%
Poor modifications (< 50)	113	42.1
Average modifications (50 to < 75)	150	56.0
Good modifications (≥ 75)	5	1.9

**Total**	**268**	**100**

A statistically significant relationship was found between knowledge and priority lifestyle modifications (*p* < 0.001) as shown in Table 6.

**TABLE 6:** Relationship between knowledge and various categories of lifestyle modifications (LSM).

Categories of LSM	Knowledge					
	Poor		Average		Good	
	*n*	%	*n*	%	*n*	%
Poor	70	55.6	41	30.8	2	22.2
Average	53	42.0	90	67.7	7	77.8
Good	3	2.4	2	1.5	-	-

**Total**	**126**	**100.0**	**133**	**100.0**	**9**	**100.0**

Note: Fisher Exact test – *p* = < 0.001.

LSM, lifestyle modifications.

## Discussion

In this study, 78.3% of the individuals with dyslipidaemia were above the age of 50 years, which is consistent with prior African and local epidemiological studies of dyslipidaemia patients.^2,11,14,15,22,26^ The mean age in this research was 60.1 years, which is consistent with the risk factors for dyslipidaemia, which include rising age.^7^

Many of the participants (70.2%) were females, with distributions similar to other South African research.^2,11,14^ According to several studies, menopausal women over the age of 45 years have the highest prevalence of dyslipidaemia; this is because of a combination of reasons, including hormonal changes and poor weight control.^25^

The findings of this research are similar to those of earlier studies, where the majority of individuals were overweight or obese, notably with central obesity, which was not evaluated in this study.^2,14,22,26^ Nearly 45% of individuals were overweight and 28% were obese, giving a total of 73% with a BMI of more than 25 kg/m^2^. According to a 2016 study by Stats SA, women in South Africa made up 63% of the overweight and obese population, compared to 31% of men.^27^ A high BMI was found to be a valid indicator of dyslipidaemia in an African meta-analysis as well as in the HAASLI trial conducted in Mpumalanga, South Africa.^14,15,22^ Obesity causes deregulation of lipoprotein metabolism, resulting in dyslipidaemia.^28^ According to the Look AHEAD randomised trial, an intentional weight loss of about 9% of initial body mass results in a reduction in the number of medications taken for type 2 diabetes and hypertension, as well as an improvement in the lipid profile, such as an increase in HDL-C and a decrease in triglyceride levels.^29^

In contrast to studies conducted in Kenya, where the majority of participants had only completed basic (primary) school, and the HAALSI study in Mpumalanga, South Africa, where education was also a predictor of developing dyslipidaemia, many participants in this study (73.1%) had not completed high school.^14,22^ Participants in this study largely consisted of people from a low socio-economic background which is documented in literature as a determinant of health. Currently, 31% of the population depend on social assistance, with persons over the age of 60 making up most of this group.^27^

In this current study, 13% of the participants were smokers. This is slightly more than the 10% self-reported smokers in Turbo County in Kenya.^22^ The findings of both studies contrast with the high frequency of 31% of smokers found in a 2010 study in Beijing.^16^ Smoking is one of the most major modifiable factors in the development of cardiovascular disease; smoking reduces HDL-C, while raising total cholesterol and LDL-C.^21,30^ Cigarette smoking leads to low HDL-C by altering the lipid-transporting enzymes and hepatic activity, which results in HDL-C metabolism dysregulation and impacts HDL-C sub-fraction distribution. It also causes oxidative modifications of HDL-C, which causes dysfunctional HDL; hence, HDL loses its atheroprotective properties in smokers.^30^ Also note that smokers have a more than 100% chance of developing dyslipidaemia.^15^ This in part explains the increased risk of cardiovascular disease in smokers.^30^ Therefore, complete cessation of all types of smoking, including cigarettes, e-cigarettes, and vaping is recommended.^7,24^ A significant smoking history of more than 10 pack years was present in 63% of smoking participants in this study, while 25.6% of those who smoke have never thought about stopping smoking and none of them have taken steps to stop.

Many participants (92.2%) seen in this study at Phedisong 4 CHC had concomitant hypertension, either alone or in combination with other cardiometabolic disorders, including diabetes mellitus (31.0%). The prevalence of hypertension in dyslipidaemic patients in this study is comparable to the data in the systematic review and meta-analysis of determinants of dyslipidaemia in African countries, as well as the 95% prevalence identified in a 2022 South African multi-centre study.^2,15^ The likelihood of acquiring dyslipidaemia was shown to be enhanced by disorders including hypertension and diabetes mellitus, which is consistent with the fact that both of these conditions have been linked to the development of dyslipidaemia.^7,9^ Only 3.4% of patients had established cardiovascular disease (any established cardiac condition grouped under cardiovascular diseases but excluding hypertension); this could be attributed to the context of the study, as patients with established cardiovascular illness receive hospital-based management and monitoring.

About half of the participants had average knowledge, and few participants had good knowledge (3.4%). Poor knowledge of dyslipidaemia in this study was comparable to the Bangladeshi study but lower than the Saudi Arabian study.^31,32^ Participants with good knowledge were very few for patients with a chronic condition that has serious consequences and requires intense patient participation in the management. Other international studies have concluded that patients who had knowledge of their disease profile and cardiovascular risk factors were more likely to reach their lipid profile targets compared to those with poor knowledge, and those with poor knowledge of the nature of disease, risks, and the benefits of long-term treatment had a negative impact on treatment adherence.^33^

The majority of the patients in this study (57.8%) knew the causes of dyslipidaemia. In the Saudi Arabian study, 56% of patients were reported to have poor knowledge of the cause.^32^ Forty-four patients in this study thought that being obese definitely means that patients have dyslipidaemia. Obesity is a known risk factor, and according to the literature, up to 70% of obese patients have dyslipidaemia.^28^ However, 56.3% knew that obesity increases the chances of developing dyslipidaemia.

The most important benefit of reducing the high cholesterol level or managing dyslipidaemia is the reduction of cardiovascular disease risk, especially atherosclerotic cardiovascular disease such as coronary artery disease, myocardial infarction and strokes, which are well-documented. However, 58.5% of the participants did not know about these positive outcomes of having a lipid profile within the correct range, which is lower than another international study.^17^ Even with this lack of knowledge, the majority (73.8% and 91.4% in the two studies, respectively) were aware of the negative outcomes of dyslipidaemia, especially hypertension and heart attacks. Similarly, 71% of participants in the Indian study were aware that dyslipidaemia was a risk factor for cardiovascular disease.

There was good self-reported adherence to statins in this study, where 87% were adherent to treatment; however, other lifestyle practices were lacking compared to taking of medication.

Only 56% of respondents said that they ate vegetables every day – more than double of the Saudi Arabian study.^32^ Over half of the patients exercised (assessed as over 30 minutes per session for more than 3 days per week), which is comparable to the African and international studies.^22,32^ Patients mainly reported walking as the main form of exercise, but this was not quantified in this study.

Participants in this study were not objectively aware that there are significant lifestyle changes to be employed at home to reduce lipid levels, with only a 38.8% awareness while majority of them had actually made those lifestyle changes seen in their responses (66.3%). This might be related to obeying health providers’ instructions without fully comprehending the rationale behind them and majority of the participants had concomitant lifestyle related conditions which contribute to improved lifestyle changes.

Most of the patients (67%) believed that they had enough information to assist them to make the necessary lifestyle modifications and 56% displayed average practice of lifestyle changes; however, 47% displayed poor knowledge. The relationship between knowledge and practices was quite significant (*p* < 0.001). This study however showed that poor knowledge did not translate to poor practices.

### Study limitations

The current study was conducted in one of the primary healthcare facilities in Pretoria, South Africa, and only patients who visited the facility during the 3 months of data collection were considered; hence, it is difficult to generalise the study’s findings to the entire country. A larger study with a larger sample size that includes people from various cultures and races, with differing eating habits, physical activities, and other variables could help in presenting an exhaustive summary of the problem in the country. Intervention was not considered nor the assessment of the outcomes, and the study considered only patients on statins for dyslipidaemia.

## Conclusion

This study found that participants’ lack of information about dyslipidaemia did not affect their behaviour because more than half of them practiced lifestyle adjustments, which can be related to the study sample of over 90% of individuals with concomitant hypertension diagnosis. Ongoing education on dyslipidemia should be addressed through a variety of venues, including health education at different health institutions.
